# “Snow globe sign” in malignant ascites and pleural effusions: a helpful sonographic finding on endoscopic ultrasound

**DOI:** 10.1055/a-2779-7409

**Published:** 2026-02-03

**Authors:** Mohamad Aghaie Meybodi, David L. Diehl, Harshit S. Khara, Amitpal S. Johal, Sara E. Monaco, Bradley D. Confer

**Affiliations:** 1 21599 Department of Gastroenterology, Hepatology and Nutrition, Geisinger Medical Center, Danville, Pennsylvania, United States; 2 21599 Department of Laboratory Medicine, Anatomic Pathology, Geisinger Medical Center, Danville, Pennsylvania, United States

**Fig. 1:**
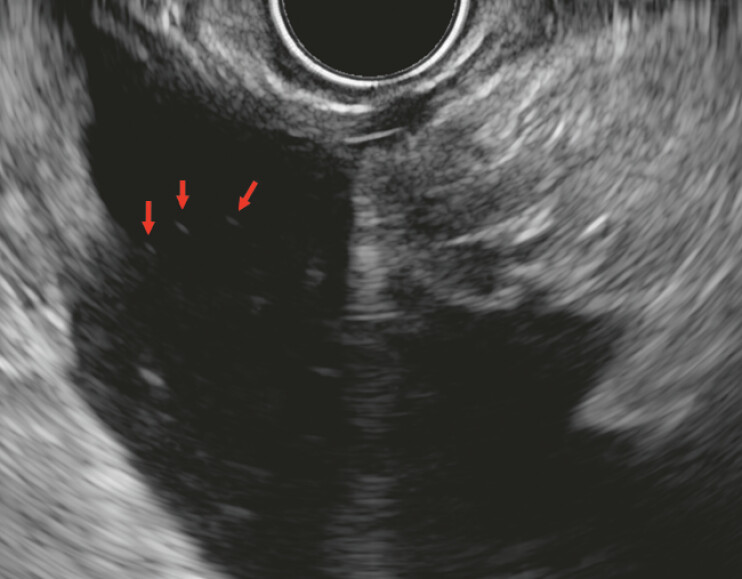
‘SGS’ in malignant ascites. Red arrows indicate hyperechoic tiny foci without an acoustic shadow floating within the ascites fluid representative of the SGS. Cytology confirmed malignant ascites secondary to pancreatic adenocarcinoma. SGS, snow globe sign.

Endoscopic ultrasound (EUS) fine needle aspiration or biopsy is widely used for the diagnosis and staging of cancer, particularly gastrointestinal and pancreaticobiliary malignancies 1 2 . EUS allows for detailed visualization and precise sampling of tissues, maximizing the accuracy of diagnosis. The presence of ascites in association with malignancy is correlated with worse prognosis 3 . Ascites or pleural effusions can be easily sampled under EUS guidance, with variable cytologic yields for malignancy of 30–60% 4 . In patients undergoing EUS for the diagnosis or staging of malignancy, we have occasionally observed the appearance of floating hyperechoic foci without acoustic shadowing within otherwise anechoic intraperitoneal or pleural fluid ( Fig. 1 ). We have termed this the ‘snow globe sign’ (SGS) ( Video 1 ). When present, it appears to be highly correlated with cytologically positive results for malignancy in the fluid.

‘SGS’ in malignant ascites or pleural effusions. SGS, snow globe sign.Video 1

A total of 56 patients (mean age 67.73 ± 13.54 y and 46.4% female) were included in the study. Of these, 54 (96.4%) had ascites and 2 (3.6%) had pleural effusions. EUS revealed the presence of the SGS in 22 patients. Cytology from fluid analysis was positive for malignancy in 20 (86.4%) of these patients, 19 with ascites and 1 with pleural effusions. The sensitivity and specificity of the SGS in diagnosis malignancy was 95 and 91.7%, respectively. In contrast, 34 patients did not exhibit the SGS, and cytology was positive in only one (2.9%) of these cases, resulting in a negative predictive value of 97.1% ( Table 1 ). The presence of the SGS was significantly associated with malignant cytology results ( *p* < 0.001).

**Table:** **Table 1** Diagnostic performance of the ‘SGS’.

Malignancy	Positive	Negative	Total	
The SGS				
Positive	19	3	22	PPV = 86.4%
Negative	1	33	34	NPV = 97.1%
Total	20	36	56	
	Sensitivity = 95%	Specificity = 91.7%		
Abbreviations: NPV, negative predictive value; PPV, positive predictive value; SGS, snow globe sign.				

Two of three patients with a SGS but no malignancy had peritoneal infections (one with an infected pseudocyst and the other with cirrhosis and spontaneous bacterial peritonitis). Pancreatic adenocarcinoma (10 patients) and ovarian cancer (2 patients) were the most common primary malignancies.

In patients undergoing EUS, the SGS in ascites or pleural effusions is highly associated with malignancy. It is both sensitive and specific for diagnosing these conditions. Its high negative predictive value is useful for ruling out malignancy in patients with a negative result.

Endoscopy_UCTN_Code_CCL_1AB_2AC_3AH
